# Supplementary material for: A novel TRIM22 gene polymorphism promotes the response to PegIFNα therapy through cytokine-cytokine receptor interaction signaling pathway in chronic hepatitis B
Source: Microbiol Spectr. 2023 Oct 26;11(6):e02247-23. doi: 10.1128/spectrum.02247-23 (PMC10715138; doi:10.1128/spectrum.02247-23)
Supplement: Supplemental material — Tables S1 and S2; Fig. S1 to S4. [file spectrum.02247-23-s0001.docx]

**A novel TRIM22 gene polymorphism promotes the response to PegIFNα therapy through cytokine-cytokine receptor interaction signaling pathway in chronic hepatitis B**

**Long Wang**^1, 2#^, **Ni Lin**^2#^, **Yanfang Zhang**^1, 2#^, Shaoying Guo^2^, Can Liu^1, 2, 3^, Caorui Lin^1, 3^, Yongbin Zeng^1, 3^, Wennan Wu^1, 3^, Jianhui Guo^1, 3^, Chenggong Zhu^1, 2^, Fuguo Zhan^1, 3^, Qishui Ou^1, 2, 3*^, Zhen Xun^1, 2, 3*^

1 Department of Laboratory Medicine, Fujian Key Laboratory of Laboratory Medicine, Gene Diagnosis Research Center, Fujian Clinical Research Center for Laboratory Medicine of Immunology, The First Affiliated Hospital, Fujian Medical University, Fuzhou 350005, Fujian, China;

2 The First Clinical College, Fujian Medical University, Fuzhou 350005, Fujian, China;

3 Department of Laboratory Medicine, National Regional Medical Center, Binhai Campus of the First Affiliated Hospital, Fujian Medical University, Fuzhou 350212, Fujian, China.

# These authors contributed equally to this work

*** Corresponding author:** Qishui Ou, E-mail address: [ouqishui@fjmu.edu.cn](mailto:ouqishui@fjmu.edu.cn). Zhen Xun, E-mail address: xunzhen@fjmu.edu.cn.

**Table of content**

Methods 3

Supplementary Table 1 4

Supplementary Table 2 5

Supplementary Fig. 1 7

Supplementary Fig. 2 8

Supplementary Fig. 3 9

Supplementary Fig. 4 10

**Transfection efficiency of the lentiviral transduction**

Evaluation of transduction efficiency was conducted using green fluorescent protein (GFP) expressed in all lentiviruses. Cells were washed three times with PBS, and then dissociated with trypsin. After cell suspensions by DMEM, cells were transferred to suitable FACS tubes. A negative control was performed using untransfected HepAD38 cells. FITC fluorescence was analyzed by flow cytometry and expressed as a percentage.

**Supplementary Table 1 Comparison of general clinical data of 107 patients**

| **Characteristics** | **SR (*n*=77)** | **CR (*n*=30)** |
| --- | --- | --- |
| Female/Male | 23/54 | 8/22 |
| Age | 27.34 ± 5.00 | 26.37 ± 3.20 |
| HBsAg (log_10_ IU/mL) | 3.99 ± 0.65 | 4.06 ± 0.54 |
| HBeAg (log_10_ S/CO) | 2.76 ± 0.63 | 2.58 ± 0.67 |
| HBV DNA (log_10_ IU/mL) | 7.12 ± 1.01 | 7.33 ± 0.65 |
| ALT (U/L) | 263.7 ± 226.8 | 298.0 ± 263.5 |
| AST (U/L) | 112.5 ± 80.72 | 131.4 ± 132.0 |

**Supplementary Table 2 qRT-PCR primers sequences used in this study**

| **Gene(s)** | **Forward primer** | **Reverse primer** |
| --- | --- | --- |
| *GAPDH* | CCATGAGAAGTATGACAACAGCC | CCTTCCACGATACCAAAGTTG |
| *TRIM22* | GAGGTCAAGATGAGCCCACA | GCAGCTTTTCCTGACATTCC |
| *TRIM14* | CGTGCAGAAACTCAGCCAAG | GCTTCTCGGTGGCATCTTCT |
| *TRIM2* | AAGCTGATGGGACCCAAAGG | GTCCCCATTTCCTCGGCTAC |
| *TRIM9* | ACTGGGAGCTCACGGTAGAT | GTGTGCGAGTTGTTGTGCAT |
| *TRIM15* | AGGAGCACGGCGAGAAGAT | GATCCCGGTAGGGCTGAATG |
| *TRIM26* | GAGGCAGTACATTGTGGCTGA | CCCCGGCTCTTGAACTTCT |
| *TRIM31* | TCGTCTGCTGGGAAAGTCAC | TCACAAAACCAAGCCCGGAT |
| *TRIM38* | GAGAGGCGGAGTGCAAATGATA | CGCTGCTAGTCCCCTCTGTT |
| *TRIM39* | ACCAAGGCGTTTCACCTTCT | AGTAGCCAGTCTCAGGGAGT |
| *TRIM40* | GGAGAGCCAGCACCAAACC | GTCACCAGCATTCTTCAGTGTGTT |
| *TRIM46* | GTGCCCAGTGTGTCAAGAGA | GGTCCCCACCATGTCCTATG |
| *TRIM61* | AATTTGTTACGGCCCTGGCT | AGGGGCAGGGGAAACTATCA |
| *TRIM63* | CCAGGCTGCAAATCCCTACT | CACTCCGTGACGATCCATGA |
| *TRIM71* | GTTATTCACCCCGACTGCCA | GGAATCCGCCACAATGATGC |
| *preS1* | GGGTCACCATATTCTTGGGAAC | CCTGAGCCTGAGGGCTCCAC |
| *IFNL1* | GGTGACTTTGGTGCTAGGCT | TGAGTGACTCTTCCAAGGCG |
| *CCL3* | CGGTGTCATCTTCCTAACCA | GACATATTTCTGGACCCACTC |
| *CCL5* | TGCTGCTTTGCCTACATTGC | CATCCTTGACCTGTGGACGA |
| *CCL3L1* | TCTGCAACCAGGTCCTCTCTG | GGCCTCTCTTGGTTAGGAAGATGAC |
| *IL15RA* | TGGCTATCTCCACGTCCACTG | AAGTCTTCATCTCTGCTGCTGGTC |
| *TNSAF9* | CGAGGGTCCCGAGCTTTCG | GCCCATCGATCAGCAGAACATTT |
| *IL11* | TGGGGACATGAACTGTGTTTGC | TCTGGGGAAACTCGAGGGG |
| *FOS* | GGGGCAAGGTGGAACAGTTAT | AGGTTGGCAATCTCGGTCTG |
| *KLHD7B* | TGCCTGTACAGCATGGAGTG | TCGTCCCAAGCATCCTTCAC |
| *LTZS1* | GCGTCAGTAGCCTCATCTCC | CCGGTTGAGCTTCTTGAGGT |
| *C6orf15* | CCTTTGCCTGGGGAGTCTTC | ATTAGAACGGGGCAGTCGTC |
| *EEF1A2* | TCTCCAAGAATGGGCAGACG | TTGACGATCTCGTCGTAGCG |
| *IGFB6P* | GGTCTACACCCCTAACTGCG | TGGAGAAAGTTTCCGGAGGG |
| *METRNL2* | GAGGCTCCATCCAGCAAGTT | CCGATAGAGTCTGCTCACGC |
| *SNPH* | CCTGCCAGCAACACCTATGA | ACTGCACGAAGTCTGTCTGG |
| *HTRA3* | GATGCGGACGATCACACCAA | TTGGATGCCGCCTCTCTGA |
| *CCL4* | GCTAGTAGCTGCCTTCTGCT | CCACAAAGTTGCGAGGAAGC |
| *TNF* | CTTCTCGAACCCCGAGTGAC | ATGAGGTACAGGCCCTCTGA |
| *LTA* | TCTGGAGAGCAAACACGGAC | ACCACCTGGGAGTAGACGAA |
| *CXCL10* | ACTGCCATTCTGATTTGCTGC | ATGCAGGTACAGCGTACAGT |
| *CLCF1* | CCCTTTCAACGAGCCAGACT | GGCTGTAGGCCTCGTAGTTC |

**
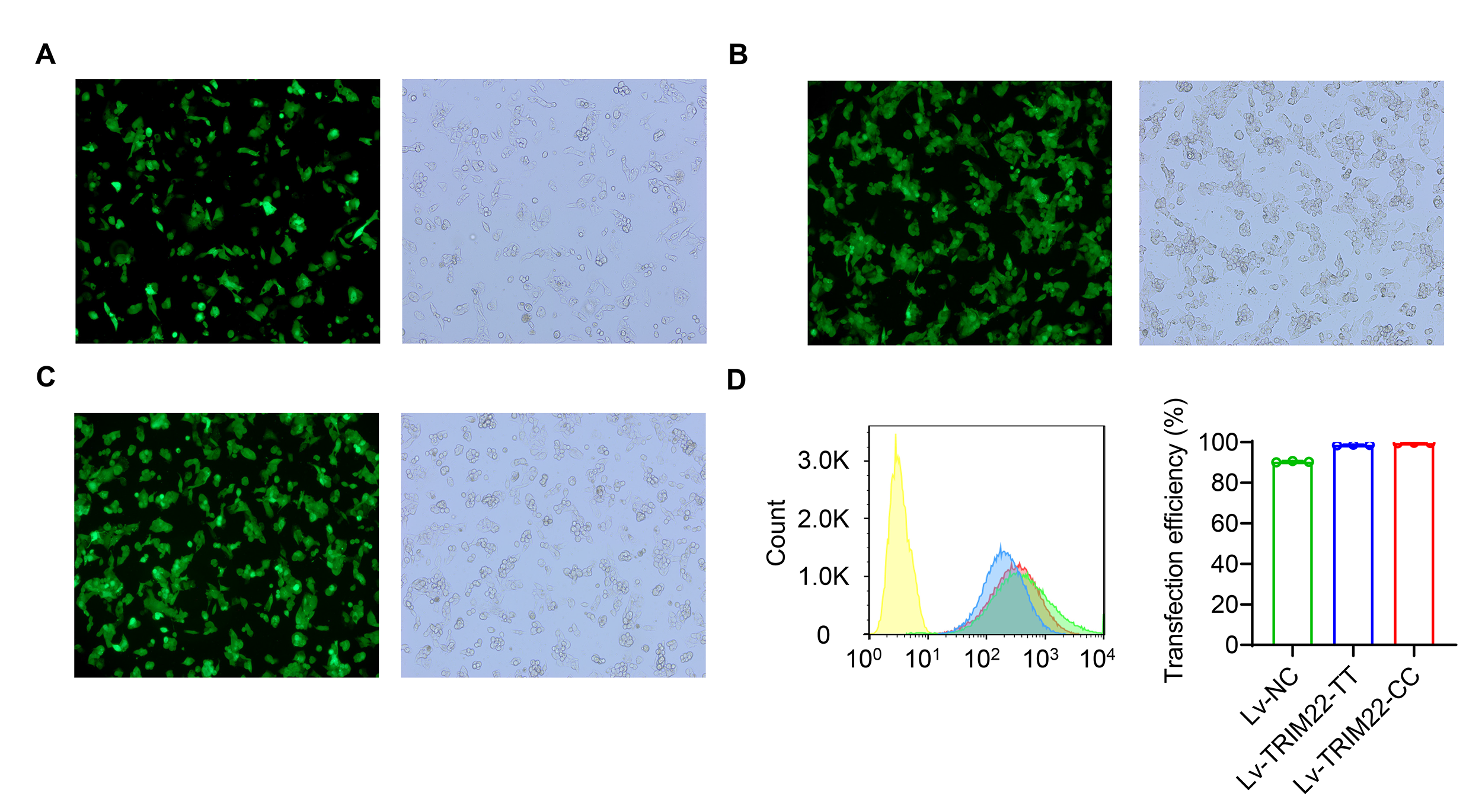
**

**Supplementary Fig. 1. Fluorescence and flow cytometry analysis of HepAD38 cells stably expressing different *TRIM22* genotypes.** **(A-C)** Fluorescence microscopy of HepAD38 cell line stably transfected with **(A)** negative control lentivirus (Lv-NC), **(B)** Lv-TRIM-TT, or **(C)** Lv-TRIM-CC. **(D)** Analysis of transfection efficiency using flow cytometry.


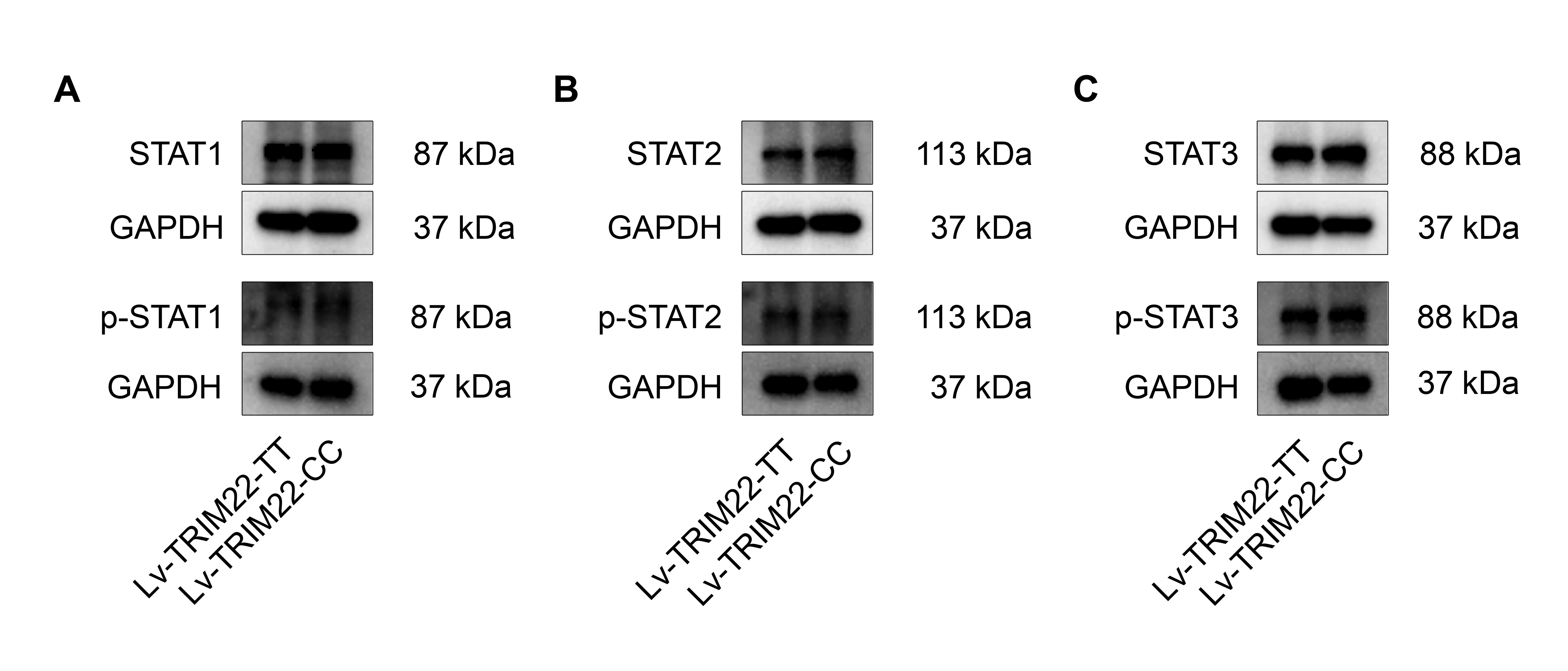


**Supplementary Fig. 2. The SNP rs10838543 genotype in *TRIM22* does not affect the JAK-STAT signaling pathway.** HepAD38 cells were stably transfected with Lv-TRIM-TT or Lv-TRIM-CC and the protein levels of **(A)** STAT1 and p-STAT1, **(B)** STAT2 and p-STAT2, and **(C)** STAT3 and p-STAT3 were analyzed by western blotting.


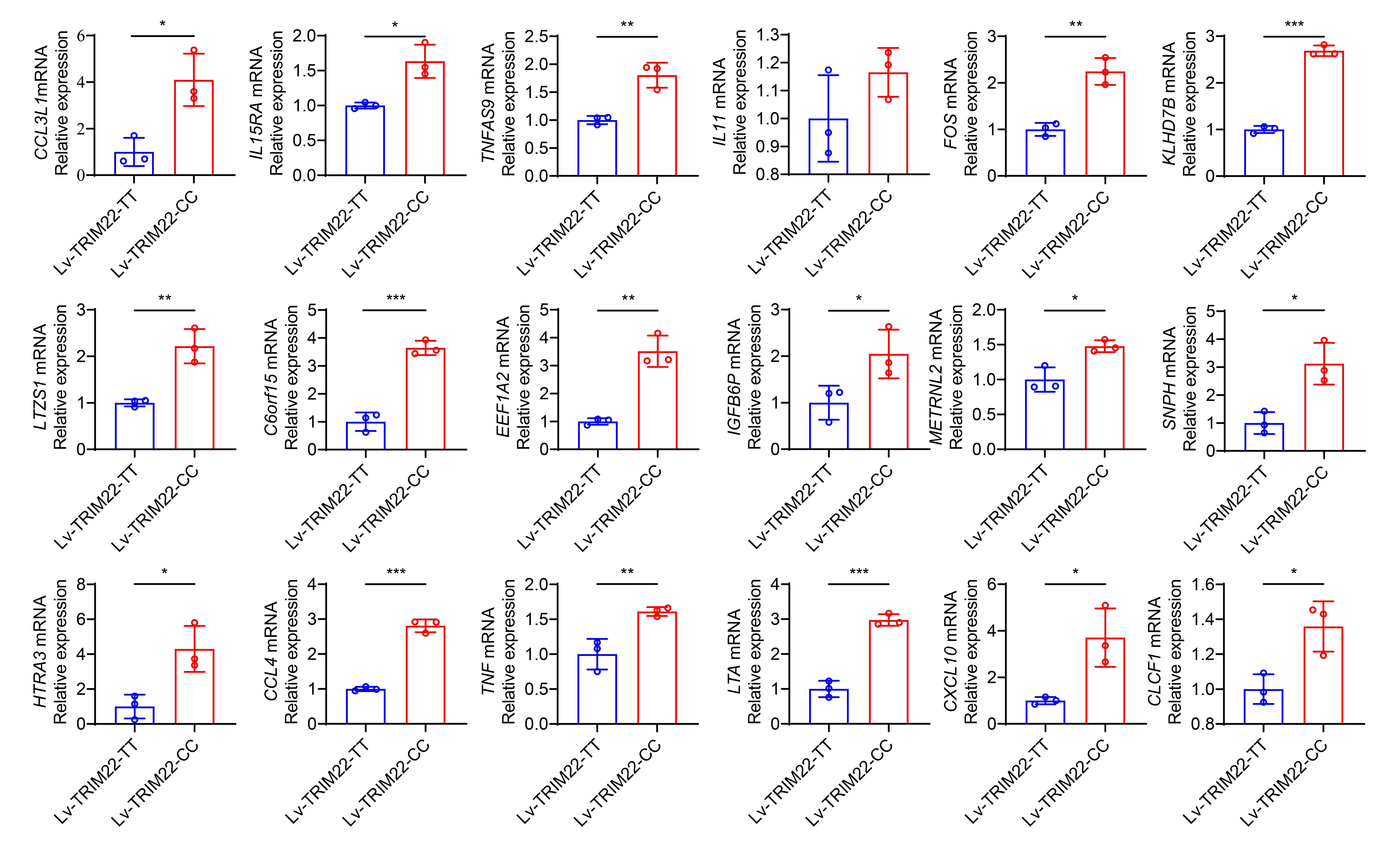


**Supplementary Fig. 3. Validation of RNA-seq results using qRT-qPCR.** Eighteen of the differentially expressed genes were randomly selected and their expression levels were analyzed by qRT-PCR using gene-specific primers. Data from a representative experiment of two or three independent replicates are shown. Unpaired Student’s *t* test, **P*<0.05, ***P*<0.01, ****P*<0.001.

**
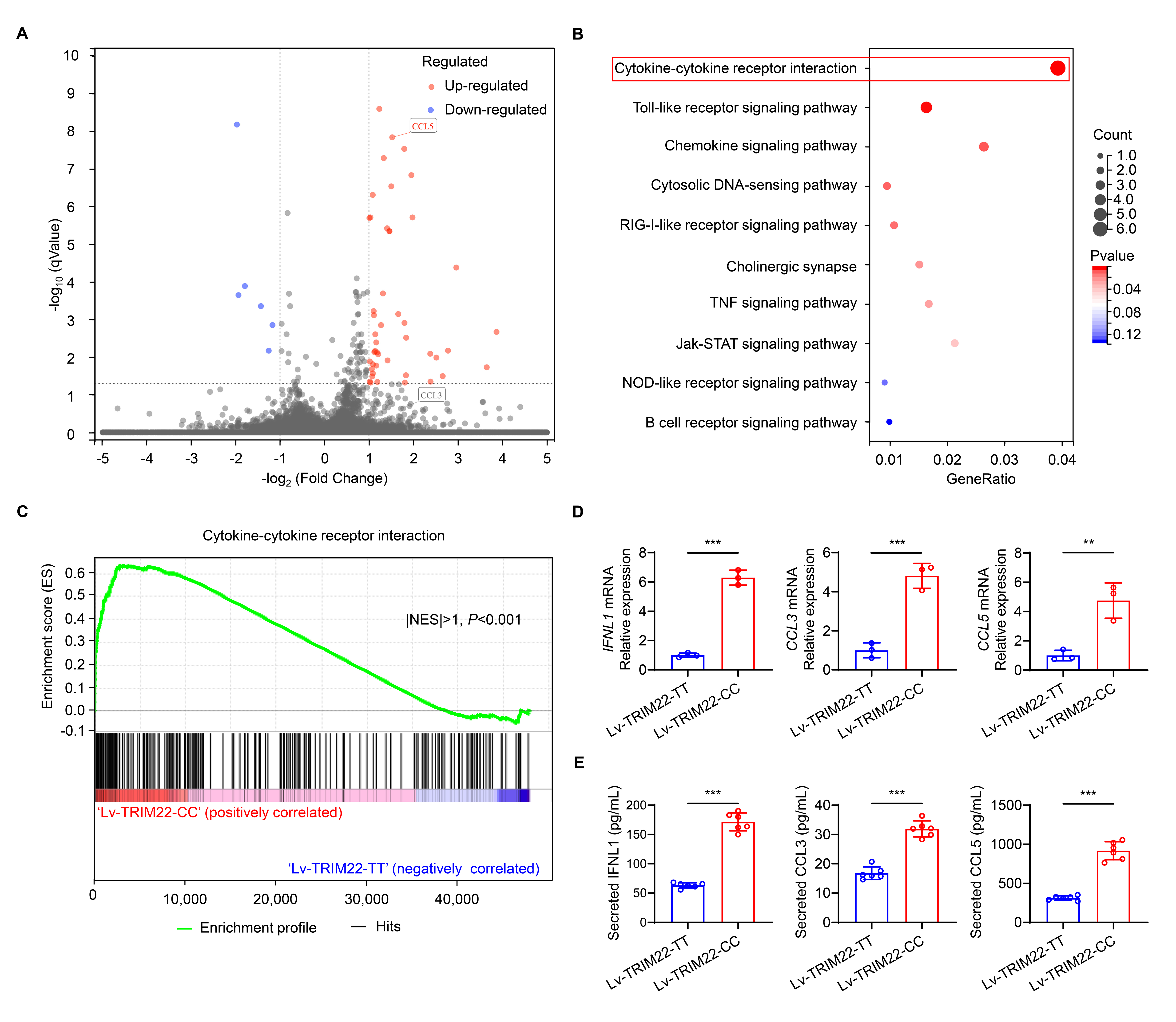
**

**Supplementary Fig. 4. The cytokine-cytokine receptor interaction signaling pathway is differentially regulated by SNP rs10838543 genotypes in *TRIM22*.** RNA-seq analysis of genes in HepAD38 cells stably expressing Lv-TRIM22-TT or Lv-TRIM22-CC (*n*=3). (**A**) Volcano plots showing differentially regulated genes; blue, downregulated; red, upregulated. (**B**) KEGG pathway enrichment analysis and **(C)** GSEA analysis of dysregulated genes identifies upregulation of the cytokine-cytokine receptor interaction signaling pathway. **(D)** qRT-PCR analysis of *IFNL1*, *CCL3*, and *CCL5* and **(E)** protein levels in HepAD38 cells stably expressing TRIM22 genotypes. Data from a representative experiment of two or three independent replicates are shown. Unpaired Student’s *t* test, **P*<0.05, ***P*<0.01, ****P*<0.001.
